# The Impact of SGLT2 Inhibitor Dapagliflozin on Adropin Serum Levels in Men and Women with Type 2 Diabetes Mellitus and Chronic Heart Failure

**DOI:** 10.3390/biomedicines11020457

**Published:** 2023-02-04

**Authors:** Alexander A. Berezin, Zeljko Obradovic, Ivan M. Fushtey, Tetiana A. Berezina, Evgen V. Novikov, Lukas Schmidbauer, Michael Lichtenauer, Alexander E. Berezin

**Affiliations:** 1 Internal Medicine Department, Zaporozhye Medical Academy of Postgraduate Education, 69000 Zaporozhye, Ukraine; 2 Department of Psychosomatic Medicine and Psychotherapy, Klinik Barmelweid, 5017 Barmelweid, Switzerland; 3 Department of Internal Medicine and Nephrology, VitaCenter, 69000 Zaporozhye, Ukraine; 4 Educational and Research Center—Ukrainian Family Medicine Training Center, Bogomolets National Medical University, 01601 Kyiv, Ukraine; 5 Department of Internal Medicine II, Division of Cardiology, Paracelsus Medical University Salzburg, 5020 Salzburg, Austria; 6 Internal Medicine Department, Zaporozhye State Medical University, 69035 Zaporozhye, Ukraine

**Keywords:** type 2 diabetes mellitus, heart failure, hemodynamics, dapagliflosin, adropin, natriuretic peptide

## Abstract

Background: adropin plays a protective role in cardiac remodeling through supporting energy metabolism and water homeostasis and suppressing inflammation. Low circulating levels of adropin were positively associated with the risk of cardiovascular diseases and type 2 diabetes mellitus (T2DM). We hypothesized that sodium–glucose linked transporter 2 (SGLT2) inhibitor dapagliflosin might represent cardiac protective effects in T2DM patients with known chronic HF through the modulation of adropin levels. Methods: we prospectively enrolled 417 patients with T2DM and HF from an entire cohort of 612 T2DM patients. All eligible patients were treated with the recommended guided HF therapy according to their HF phenotypes, including SGLT2 inhibitor dapagliflozin 10 mg, daily, orally. Anthropometry, clinical data, echocardiography/Doppler examinations, and measurements of biomarkers were performed at the baseline and over a 6-month interval of SGLT2 inhibitor administration. Results: in the entire group, dapagliflozin led to an increase in adropin levels by up to 26.6% over 6 months. In the female subgroup, the relative growth (Δ%) of adropin concentrations was sufficiently higher (Δ% = 35.6%) than that in the male subgroup (Δ% = 22.7%). A multivariate linear regression analysis of the entire group showed that the relative changes (Δ) in the left ventricular (LV) ejection fraction (LVEF), left atrial volume index (LAVI), and E/e’ were significantly associated with increased adropin levels. In the female subgroup, but not in the male subgroup, ΔLVEF (*p* = 0.046), ΔLAVI (*p* = 0.001), and ΔE/e’ (*p* = 0.001) were independent predictive values for adropin changes. Conclusion: the levels of adropin seem to be a predictor for the favorable modification of hemodynamic performances during SGLT2 inhibition, independent ofN-terminal brain natriuretic pro-peptide levels.

## 1. Introduction

During the last decades, the prevalence of type 2 diabetes mellitus (T2DM) has continued to increase [1]. According to an evaluation by WHO statistics experts, more than 422 million adults globally had DM in 2014, and this number is expected to rise 3-fold by 2035, which makes the global burden of this disease extremely challenging [1,2]. In light of this fact, several complications of T2DM, such as heart failure (HF) and cardiovascular diseases (CVD), have steadily arisen in the general population [3,4]. In fact, T2DM is a powerful risk factor in any phenotype of HF for all-cause and cardiovascular (CV) mortality [5,6]. Moreover, the increase in HF with a preserved ejection fraction (HFpEF) worldwide mainly relates to the increase in the prevalence of T2DM and other metabolic conditions, including abdominal obesity and CVD [7]. Despite a tendency of new cases of HF with reduced ejection fractions (HFrEF) to decline in the majority of developed countries due to the wide implementation of novel diagnostics and treatment standards, the global prevalence of HF seems to have increased as result of the impact of conventional CV factors, such as T2DM as well as gender- and age-related causes [8].

It has been considered that the main underlying pathogenetic mechanisms of the development of diabetes-related vascular complications and adverse cardiac remodeling leading to HF exhibit strict resemblance [9]. Impaired myokine production, along with microvascular and inflammatory activation due to myocardium and skeletal muscle-adipose tissue axis dysfunction, mediates the accumulation of collagen in the extracellular matrix of the myocardium, accelerating atherosclerosis and worsening endothelial function, oxidative stress, and mitochondrial dysfunction, leading to adverse cardiac remodeling, reduced angiogenesis, altered coronary reserve, skeletal muscle weakness, and myopathy [10,11]. All these play a pivotal role in the development of HF in T2DM and the response to conventional therapies [10,11]. Previous studies have shown that, being produced by skeletal muscles as well as the myocardium and adipose tissue, myokines can provide potentially far-reaching effects on non-muscle tissues, including vascular and kidney tissue [12]. Indeed, the autocrine, paracrine, and endocrine actions of myokines include the regulation of energy expenditure, insulin sensitivity, the oxidation of free fatty acid, adipocyte browning, glycogenolysis, glycogenesis, water and electrolyte homeostasis, and bone metabolism [13]. Many myokines (adropin, apelin, isirin) are organ-protective molecules that stimulate autophagy, reduce inflammation, inhibit endoplasmic reticulum stress, and improve reparation, whereas others (myostatin) induce maladaptive mechanisms in the myocardium and skeletal muscles [14,15]. Recently, several clinical studies confirmed the predictive values of irisin and apelin in HF patients with T2DM [16,17,18]. Less known, the role of dynamic changes in adropin in HF patients with T2DM in connection to cardiac function depends on gender, because the accumulation of adipose tissue and skeletal muscle mass differs between male and female populations.

Adropin is a unique multi-functional circulating protein that is encoded by the energy-homeostasis-associated gene (*Enho*) and produced by the myocardium, skeletal muscles, liver, adipocites, as well as the brain, lung, kidney medulla, and circulating peripheral blood mononuclear cells [19]. Adropin acts through the G protein-coupled (GPR19) receptor, regulates energy homeostasis and lipid metabolism, suppresses inflammation and water consumption, stimulates diuresis, prevents insulin resistance, and impairs glucose tolerance [20,21]. Low circulating levels of adropin were found in patients with abdominal obesity, T2DM, atherosclerosis, myocardial infarction, and HF [22,23,24,25]. Furthermore, adropin concentrations are correlated with age, gender, and the number of CV and metabolic risk factors, and increase in humans after gastric bypass surgery and after reaching good glycemic control [26]. Several previous meta-analyses showed that decreased adropin levels might play a crucial role in the development of CVD and HF [27,28].

Currently, sodium–glucose linked transporter 2 (SGLT2) inhibitors are included in the contemporary therapy of different phenotypes of HF regardless of T2DM presentation, while until now, the underlying mechanisms by which these agents are exerted, and their ability to improve clinical outcomes and hemodynamics, are not deeply understood. Perhaps the cardiac protective effects of SGLT2 inhibitors relate to their ability to enhance gluconeogenesis and ketogenesis through the activation of sirtuin-1 and its two downstream mediators–such as the proliferator-activated receptors gamma coactivator 1-alpha and fibroblast growth factor-21–as well as via supporting vasodilation and nitric oxide bioavailability [29]. Adropin intervenes in oxidative stress and modulates autophagy through the lysosome-dependent degradative pathway, which contains molecular targets for SGLT2 inhibitors. It remains uncertain whether SGLT2 inhibitors can modulate the level of adropin in peripheral circulation and thereby exert several favorable effects in HF patients with T2DM. The aim of this study was to investigate the effect of SGLT2 inhibitor dapagliflosin on the levels of adropin in males and females with T2DM with chronic HF.

## 2. Materials and Methods

### 2.1. Study Population and Design

**Figure 1:**
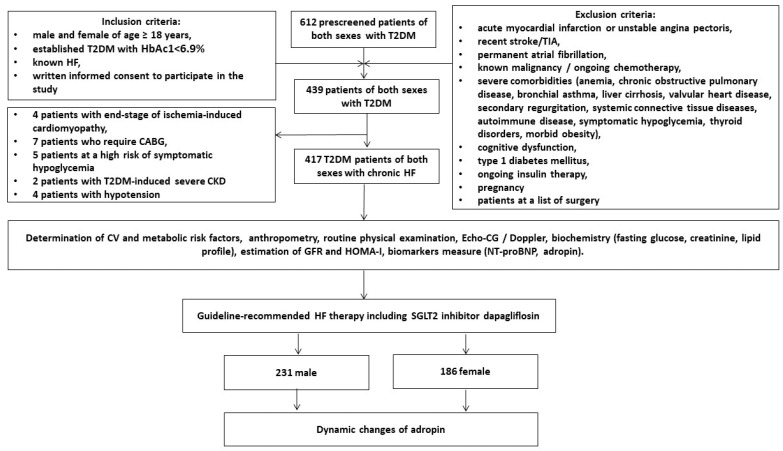
Flow chart of the study design. Abbreviations: T2DM, type 2 diabetes mellitus; CABG, coronary artery bypass grafting; CKD, chronic kidney disease; TIA, transient ischemic attack; HF, heart failure.

The study was an open-label, multicenter (VitaCenter Zaporozhye, Ukraine, EliteMedService, Zaporozhye, Ukraine and City Hospital #7, Zaporozhye, Ukraine) non-randomized cohort investigation. Patients of both sexes were included from October 2020 to July 2022 who were aged ≥ 18 years and had established T2DM with HbAc1 < 6.9%, hemodynamically stable HF (II-III NYNA functional classes), and written consent to participate in the study. The main inclusion and exclusion criteria were reported in detail in a previously published article [30]. According to these criteria, we prospectively enrolled 417 patients with T2DM and HF from the entire cohort of 612 T2DM patients. Figure 1 reports the study design and the list of procedures in detail.

The eligible patients were treated with the recommended guided HF therapy according to their HF phenotypes, including the consumption of SGLT2 inhibitor dapagliflozin at a dose of 10 mg OD orally, along with that of an angiotensin receptor-neprilysin inhibitor (ARNI)/ACE inhibitors/angiotensin-II receptor blockers (ARBs), a mineralocorticoid receptor antagonist (MRA), and beta-blockers. As a basic antidiabetic medication, metformin was administered, the dose of which was personally adjusted to the patients at the beginning of the study so that the patients had a criterion of HbAc1 < 6.9%. The lifestyle modification program was adjusted to the patients with T2DM before their enrollment and entry in the study. Blood-pressure-lowering agents were administered to maintain office BP < 140/90 mmHg and/or average daily BP < 130/80 mm Hg. Diuretic management was performed with loop diuretics, which were given in individually adjusted daily doses according to the current clinical situation, the dynamics of body mass and edema, daily diuresis, and when clinical signs of fluid retention were found.

Lipid-lowering agents were administered in the patients with dyslipidemia, established coronary artery disease, or chronic kidney disease who did not show conventional contraindications. Antiplatelet drugs and oral anticoagulants were used when needed to prevent CV complications and/or systemic/local thromboembolic events. The observation period was 6 months.

### 2.2. Methods

#### 2.2.1. Anthropometric Assessment

The standard anthropometric examinations included height, weight, waist circumference, hip-to-waist ratio (WHR), and body mass index (BMI), which was calculated as weight (kg)/height (m)^2^ for each person.

#### 2.2.2. Determination of HF, CV Disease, CV Risk Factors, and Clinically Significant Co-Morbidities

A routine clinical assessment included interviews, reviews of medical records, standard physical examinations to poll clinically significant data on a background profile, CV risk factors, previous and current medication use, symptoms of HF and T2DM, and HF functional status, according to the New York Heart Association (NYHA). Conventional factors of CV risk including hypertension, dyslipidemia, and a smoking habit were evaluated in compliance with the current guidelines of the European Society of Cardiology (ESC) [31]. T2DM, stable coronary artery disease, chronic kidney disease, and HF were diagnosed according to the current recommendations [32,33,34,35,36].

#### 2.2.3. Echocardiography Examination

A standard transthoracic B-mode echocardiography/Doppler examination was carried out with commercially available ultrasound systems, comprising “GE Medical Systems” (General Electric, Freiburg, Germany), “Aplio 400” (Canon Medical Systems, Tochigi, Japan), and “Vivid E9” (General Electric Vingmed Ultrasound AS, Horten, Norway), in accordance with the current guidelines of the American Society of Echocardiography/European Association of Cardiovascular Imaging [37]. The echocardiographic Doppler assessments were focused on LV systolic and diastolic functions and LV hypertrophy (LVH) [38]. Cardiac volumes, left ventricular (LV) ejection fractions (LVEF), and left atrial volume indices (LAVI) were measured using the Simpson method. The LV end-diastolic volume (LVEDV) and LV end-systolic volume (LVESV) were normalized to the body surface area (BSA) and given as the LVEDV index (LVEDVi) and LVESV index (LVESVi). LVH was determined by the conventional echo criteria (LV mass/body surface area ≥125 g/m^2^ in males or ≥110 g/m^2^ in females). The early diastolic wave velocity (E) and mitral annular early diastolic velocity, given as averaged septal and lateral e` (e`) and E/e’ ratios, were determined by a pulsed-wave Doppler and a spectral tissue Doppler obtained from the apical 4-chamber view.

#### 2.2.4. Estimating Glomerular Filtration Rate and Insulin Resistance Evaluation

The glomerular filtration rate (GFR) was calculated using the CKD-EPI formula [39]. Insulin resistance was evaluated with the Homeostatic Assessment Model of Insulin Resistance (HOMA-IR) using the conventional equation [40].

#### 2.2.5. Blood Sampling and Biomarkers’ Measurement

Fasting blood samples were collected from an antecubital vein and then they were placed in silicon tubes. Within 30 min of blood sampling collection, each sample was transferred to the laboratory. The plasma was received after centrifugation for 15 min at 1600× *g* at 4 °C. Then, the polled serum aliquots were immediately stored in a refrigerator at ≤−70 °C until further analysis. We routinely used Roche P800 analyzer (Roche, Basel, Switzerland) to measure the fasting levels of fasting glucose, insulin, glycosylated hemoglobin (HbA1c), total cholesterol (TC), low-density lipoprotein (LDL-C) cholesterol, high-density lipoprotein (HDL-C) cholesterol, and triglycerides (TG). The serum levels of adropin and NT-proBNP were detected using commercially available ELISA kits (Elabscience, Houston, TX, USA) according to the manufacturer’s instructions.

#### 2.2.6. Definitions of End-Point

The primary end-point was defined as a 6-month change in serum levels of adropin after the beginning of dapagliflozin administration.

#### 2.2.7. Statistical Analysis

v. 23 Statistical Packages for Social Sciences (SPSS; IBM, Armonk, NY, USA) software and v. 9 GraphPad Prism (GraphPad Software, San Diego, CA, USA) software were used for statistical analysis. The Kolmogorov–Smirnov test was performed to determine the normality of the data distribution. Continuous variables with normal distribution were characterized by the mean (M) ± the standard deviation (SD), whereas continuous, non-normally distributed variables were specified by the median (Me) and interquartile range (IQR). Categorical variables were reported as frequencies and percentages. To compare the categorical variables between the female and male subgroups, we performed a Chi-square family test. We used Student’s *t*-test for comparisons of continuous data, which are normally distributed. Differences in the parameters at the baseline and over a 6-month treatment period were compared by paired Student *t*-tests or the Wilcoxon signed-rank test. Links between changes in adropin levels with clinical status, cardiac performances, and circulating biomarkers were evaluated by univariate linear regressions. The variables of *p* < 0.1 in the univariate analysis were included in the automatic forward stepwise variable selection procedure for multivariate regression tests. Tukey’s honestly significant difference test for post hoc multiple testing was applied. Thye determination of the inter- and intra-observer reproducibility of adropin concentrations in the peripheral blood of 60 randomly selected patients was performed by estimating the intra-class correlation coefficient. Differences were considered significant at the level of statistical significance *p* < 0.05.

## 3. Results

### 3.1. Patients’ Characteristics

Table 1 reports the baseline clinical characteristics, echocardiographic features, and biomarkers of T2DM patients with known HF. The patients’ mean age was 53 (41–64) years, the mean body mass index (BMI) was 25.8 kg/m^2^, the mean waist circumference was 85.1 cm, and the mean waist-to-hip ratio (WHR) was 0.85 units. The coexisting conditions and risk factors were composed of dyslipidemia (83%), arterial hypertension (84.4%), stable coronary artery disease (33.8%), smoking (40.3%), abdominal obesity (42.9%), microalbuminuria (32.4%), left ventricular hypertrophy (80.1%), chronic kidney disease (26.9%), and atrial fibrillation (13.7%). Eligible patients were in the II (67.6%) and III (32.4%) HF NYHA classes and were qualified as having HFpEF (31.7%), HFmrEF (33.6%), and HFrEF (34.8%). All patients were hemodynamically stable. The average of the LV ejection fraction (LVEF) was 46% (39–54%), the LAVI was 43 mL/m^2^, and the E/e’ ratio was 13.5 units. The mean levels of HbA1c, creatinine, NTproBNP, and adropin were 6.59 ± 0.02 %, 108.6 ± 8.5 µmol/L, 2615 (1380–3750) pmol/mL, and 237.40 (190.50–275.30) pg/mL, respectively. We did not notice significant differences between subgroups in age, BMI, waist circumference, WHR, presentations of dyslipidemia, hypertension, stable CAD, CKD 1–3 grades, atrial fibrillation (AF), blood pressure (BP), eGFR, HOMA-IR, HbA1c, creatinine, lipid profiles, or concomitant medications.

On the contrary, abdominal obesity (*p* = 0.046) was detected more frequently in the female subgroup than in the male subgroup. Additionally, the HFpEF and HF NYHA class II were detected more frequently in the female subgroup (*p* = 0.044 and *p* = 0.040, respectively), whereas the HFrEF and HF NYHA class III were found less often in the male subgroup (*p* = 0.042 and *p* = 0.040, respectively). Aligned with this, the male subgroup demonstrated significantly higher values for LV volume, LVEDVi, LVESVi, LVMMI, and LAVI, and marginally lower LVEFs than the female subgroup. Furthermore, patients from the male subgroup had higher levels of NT-proBNP (*p* = 0.020) and lower levels of adropin (*p* = 0.010) than the female subgroup did. Liver enzymes were detected in the circulating blood before and after SGLT2 inhibitor administration, but we did not find clinically significant changes in their concentrations in both groups. Evidence of ketosis was not found in urine samples in any patients in the study at the baseline and at the beginning of the study.

### 3.2. Spearman’s Correlation between Circulating Levels of Adropin and Other Parameters

In the entire group of patients, we found positive correlations of adropin levels with LAVI (r = 0.32; *p* = 0.001), NYHA HF class II (r = 0.30, *p* = 0.012), BMI (r = 0.29, *p* = 0.010), and eGFR (r = 0.31; *p* = 0.001), and negative correlations of adropin levels with LVEF (r = −0.34; *p* = 0.001) and NT-proBNP (r = −0.36; *p* = 0.001), but these were not associated with the HOMA index. There were no significant correlations between the baseline levels of NT-proBNP and adropin and concomitant medications. In the female subgroup, the negative correlation between adropin and NT-proBNP was more profound (r = −0.40; *p* = 0.001) than that in male subgroup (r = −0.36; *p* = 0.001). Therefore, in the female subgroup, we found a significant correlation of adropin with LVMMI (r = −0.38; *p* = 0.001) and microalbuminuria (r = 0.32; *p* = 0.001), whereas in the male subgroup, there were no significant associations along these parameters (r = −0.11; *p* = 0.216 and r = 0.142; *p* = 0.223, respectively).

### 3.3. Changes in Serum Levels of Adropin in Comparisson with NT-proBNP during Dapagliflozin Administration in Males and Females

**Figure 2:**
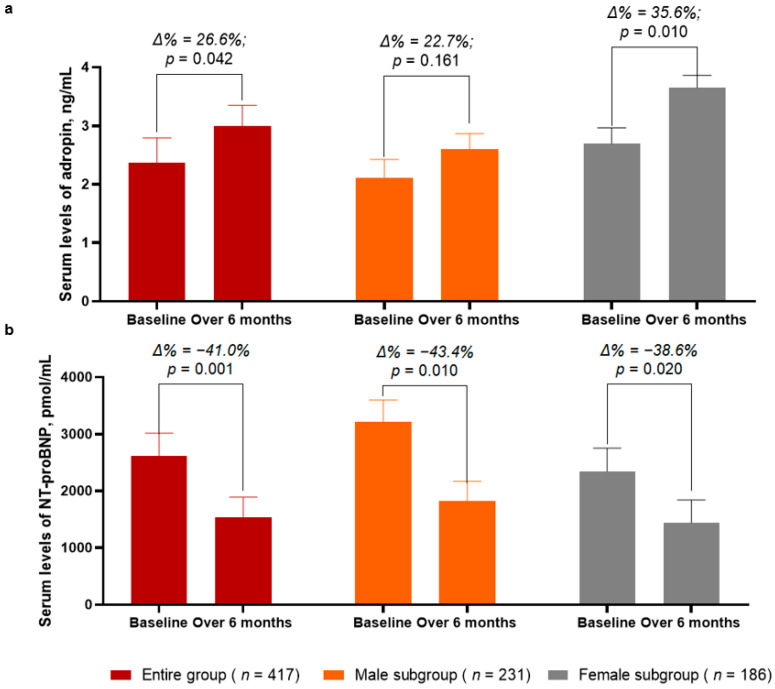
Bar graphs at baseline and 6 months after administration of SGLT2 inhibitor dapagliflozin, showing dynamics of serum levels of adropin (**a**) and NT-proBNP (**b**). Abbreviations: Δ%, respective percentage of changes of parameters.

Over a 6-month period after the initial prescription of SGLT2 inhibitor dapagliflozin, the levels of adropin in the entire group demonstrated a significant increase of up to 26.6% (from 2.37 [25–75% IQR = 1.91–2.75] ng/mL to 3.00 [25−75% IQR = 2.68–3.36] ng/mL, *p* = 0.042) (Figure 2a). In the female subgroup, the increase in the circulating levels of adropin was sufficiently higher (Δ% = 35.6%, from 2.69 [25–75% IQR = 2.31–2.99] ng/mL to 3.65 [25–75% IQR = 3.40–3.89] ng/mL, *p* = 0.010)when compared with those of the male subgroup (Δ% = 22.7%, from 2.11 [25–75% IQR = 1.90–2.37] ng/mL to 2.60 [25–75% IQR = 2.07–3.21] ng/mL, *p* = 0.161).

In the entire group, the circulating levels of NT-proBNP significantly decreased from 2615 (1380–3750) pmol/mL to 1542 (25–75% IQR = 970–2075) pmol/mL (Δ% = −41.0%, *p* = 0.001) between the baseline and 6 months after dapagliflozin administration (Figure 2b). Although in both subgroups the levels of NT-proBNP were found to be significantly decreased, in the male subgroup, the decrease of NT-proBNP was a little bit better (Δ% = −43.4%, from 3218 [25–75% IQR = 2870–3689] pmol/mL to 1820 [25−75% IQR = 1440–2310] pmol/mL, *p* = 0.010) when compared with that of the female subgroup (Δ% = −38.6%, from 2344 [25–75% IQR = 1930–2750] pmol/mL to 1440 [25–75% IQR = 1015–1870] pmol/mL, *p* = 0.020).

### 3.4. Changes in Clinical Performances and Hemodynamics Parameters during Dapagliflozin Administration

Table 2 reports the dynamics of the clinical data, hemodynamics characteristics, and biomarkers in the patients during SGLT2 inhibitor dapagliflozin administration.

There were no significant changes in BMI in the entire group as well as in the male and female subgroups. However, the proportion of HF patients in the II and III NYHA classes demonstrated sufficient changes over the treatment period. In the entire population, there was a significant increase in the amount of HF patients with class II HF NYHA and a decrease in those who had class III HF NYHA. Along with this, the number of females whose HF functional class improved was significantly higher compared to that of males, while the total number of patients of both genders with HF functional class III was sufficiently reduced. In addition, we found that in the entire group, the left ventricular end-systolic volume (LVESV), left ventricular myocardial mass index (LVMMI), left atrial volume index (LAVI), and early diastolic blood filling to longitudinal strain ratio (E/e’) significantly decreased, whereas there were no significant changes in the left ventricular end-diastolic volume (LVEDV) and left ventricular ejection fraction (LVEF). We noticed that the LVESV, LVMMI and LAVI decreased only in the female subgroup, whereas E/e’ significantly decreased in all sexes. Along with this, there were no changes in the profile of conventional biomarkers (eGFR, fasting glucose, creatinine and HbAc1) in eligible patients regardless of gender.

### 3.5. Association of Adropin Changes with Clinical Parameters, Hemodynamics Performances and Circulating Biomarkers during Administration of SGLT2 Inhibitor Dapagliflozin

To evaluate the plausible associations of adropin dynamics with other variables after the administration of dapagliflozin in gender-related aspects, we performed univariate and multivariate linear regression analyses in the entire patient group and then in the subgroups of males and females (Table 3).

An unadjusted univariate linear regression analysis showed that the BMI at the baseline (*p* = 0.046) and relative changes (Δ) of LVEF (*p* = 0.040), LAVI (*p* = 0.001), LVMMI (*p* = 0.012), E/e’ (*p* = 0.001), NYHA class (*p* = 0.042), and NT-proBNP (*p* = 0.012) were significantly associated with the adropin levels during SGLT2 inhibitor dapagliflozin administration, whereas age, abdominal obesity, and ΔBMI were not. Multivariate linear regression indicated that only ΔLVEF (*p* = 0.046), ΔLAVI (*p* = 0.001), and ΔE/e’ (*p* = 0.001) remained strongly associated with the changes in adropin levels, whereas ΔBMI (*p* = 0.052), ΔLVMMI (*p* = 0.052), and ΔNT-proBNP (*p* = 0.050) exhibited borderline significance in this matter.

In the male subgroup, the BMI at the baseline (*p* = 0.046), ΔLAVI (*p* = 0.042), ΔE/e’ (*p* = 0.012), and ΔNT-proBNP (*p* = 0.014) were found to be predictors for changes in adropin levels in the peripheral circulation in the unadjusted univariate linear regression analysis, whereas multivariate linear regression indicated that the only ΔE/e’ retained its independent predictive potency (*p* = 0.014).

In the female subgroup, abdominal obesity (*p* = 0.048), BMI at the baseline (*p* = 0.041), ΔBMI (*p* = 0.042), ΔNYHA class (*p* = 0.042), ΔLVEF (*p* = 0.040), ΔLVMMI (*p* = 0.001), ΔLAVI (*p* = 0.001), ΔE/e’ (*p* = 0.001), and ΔNT-proBNP (*p* = 0.012) were found to be predictors for adropin level changes in the unadjusted univariate linear regression. However, ΔLVEF (*p* = 0.046), ΔLAVI (*p* = 0.001), and ΔE/e’ (*p* = 0.001) exhibited their independent predictive values in multivariate linear regression.

### 3.6. Reproducibility of Adropin

We evaluated the reproducibility of adropin in comparison with NT-proBNP in the patients of both genders. The intra-class correlation coefficient for the inter-observer reproducibility of NT-proBNP was 0.88 (95% confidence interval [CI] = 0.83–0.92). The intra-class correlation coefficient for the intra-observer reproducibility of adropin was 0.91 (95% CI = 0.85–0.97). We did not find significant changes in the intra-observer reproducibility of adropin depending on the gender of eligible patients (*p* = 0.382).

## 4. Discussion

The results of the study revealed a convincing difference among the adropin levels over time for male versus female patients. Additionally, we noticed that the 6-month administration of SGLT2 inhibitor dapagliflosin was associated with NYHA HF class improvement and favorable changes in LVESV, LVEF, LVMMI, and diastolic function parameters, which corresponded to a trend of decrease in NT-proBNP and the opposite dynamic in serum levels of adropin. We noticed that the elevation of adropin concentrations in peripheral blood predicted favorable changes in cardiac remodeling performances (LVEF, LAVI and ΔE/e’) mainly in the female population of eligible patients and not in male population. We are the first to report these findings, so we believe that this particularity of SGLT2 inhibitor dapagliflosin seems to explain the innate capability of the agent to work actively in the female population, although it requires further investigation in the future.

The difference in the circulating levels of adropin in males and females may relate to adipose tissue accumulation, the mass of skeletal muscles, and dietary macronutrient intake, which seem to be different between these populations [19]. Moreover, estrogen impact on the expression of the adropin gene in the liver, adipose tissues, and skeletal myocytes is crucial for glucose and lipid homeostasis supported by adropin [10,12,13]. Perhaps, gender-related differences in adropin levels deserve to be investigated in the future in the context of the adaptive role of this protein in the regulation of energy homeostasis and cardiac remodeling in HF individuals.

We speculate that the relative changes in hemodynamics are crucially important because the use of SGLT2 inhibitors did not affect the clinical conditions and conventional parameters of cardiac remodeling, while the dynamics of LVEF and cardiac volumes might be considered a predictor for the benefits of SGLT2 inhibitors. On the other hand, changes of hemodynamics may be predictable by biomarkers, such as NT-proBNP and adropin. Previous studies showed that SLGT2i were effective in improving the prognosis of HF patients regardless of NT-proBNP, so there is a need for a new surrogate marker by which the dynamic changes of hemodynamics are considered and predicted. In addition, SGLT2 inhibitors have been shown to have a protective endothelial function and to protect the myocardial microvascular compartment in T2DM [41,42].

In fact, there is still no strong evidence of the theory that, in several populations with HF, including HFpEF, SGLT2 inhibitors exert a more profound impact on the clinical outcomes, surrogate image, and circulating biomarkers of adverse cardiac remodeling in female than in male patients [43]. Moreover, these agents demonstrated strict similarity in benefits among patients with and without atherosclerotic CV disease, a history or presentation of HF, T2DM, and chronic kidney disease [44,45,46]. There are numerous explanations for this, which partially include the difference in pre-existing comorbidity profiles, anthropomorphic characteristics, the etiology of HF, and responses to the treatment [47,48,49,50]. Another possible explanation may be the plausible role of sex-hormone-related regulation of the activity of the sodium–hydrogen exchanger (NHE)−1 co-transporter by which SGLT2 inhibitors are enabled to restore an expression of the X-linked inhibitor of apoptosis and the baculoviral IAP repeat-containing protein 5 (BIRC5), and thereby, to ameliorate cardiomyocyte cell death, prevent extracellular matrix accumulation, and suppress oxidative stress and mitochondrial dysfunction [51,52,53]. Additionally, there is an assumption that the expression of the target signaling molecules of SGLT2 inhibitors on cell surfaces may directly relate to gender and indirectly relate to an accumulation of adipose tissue, so the final effect of SGLT2 inhibitors is considered to be attributed to sex-hormone impacts [54]. Indeed, SGLT2 inhibitors, through promoting a nutrient deprivation signaling system–which is composed of Sirtuin-1, fibroblast growth factor-21, and peroxisome proliferator-activated receptor-gamma coactivator-1 alpha–are able to stimulate ketogenesis and gluconeogenesis in target cells, ultimately attenuating maladaptive cardiac remodeling, reducing microvascular inflammation and oxidative stress, and improving endothelial function [55]. There was a strong difference in the tissue expression of the main components of this system between both sexes [56]. Thus, SGLT-2 inhibitors have numerous remarkable effects on suppressing the progression of HF and preventing adverse cardiac remodeling in T2DM through multiple molecular mechanisms, part of them exhibiting gender-based activity and being under the control of autocrine signaling pathways.

In the study, we concentrated on our hypothesis, which is based on the speculation that adropin may be a promising biomarker, allowing us to identify patients with HF who show a tendency of declining levels of NT-proBNP in their peripheral blood. Although the recent trials and meta-analyses revealed that the effects of SGLT2 inhibitors in HF patients on all-cause and CV mortality did not vary by sex, T2DM duration, or the presence of CV disease [57,58,59,60], this does not mean that all HF patients will receive benefits from SGLT2 inhibitors. In fact, patients with HF with improved levels of NT-proBNP, as a result, t, may be overvalued at the risk of poor response in cardiac performances and outcomes in conventional treatment. In this connection, the surrogate and validated circulating biomarker, which illustrates well the overlapping pathogenetic mechanisms of both HF and T2DM, seems to be promising for further investigations in large clinical studies.

Although adropin was found to be a potential regulator of CV functions, playing a key protective role in the pathogenesis of HF and T2DM [60], there is a limiting number of preclinical data and clinical evidence corresponding to our hypothesis. In a cohort of patients with end-stage kidney disease, there was no evidence of a significant correlation between serum levels of adropin and LV septal thickness [61], whereas adropin negatively regulated cell proliferation through the AMP-activated protein kinase/acetyl-CoA carboxylase signaling pathway and thereby prevented hypertrophy [62]. In some studies, increased levels of adropin related to the severity of acute HF and cardiac cachexia [28,63], but in patients with chronic HF, adropin levels in the peripheral blood were sustainably low [27]. In our study, in patients with T2DM of both sexes, there was a trend of increase in the circulating levels of adropin in response to the administration of SGLT2 inhibitor dapagliflosin, accompanied by an improvement of cardiac hemodynamics performances. We did not notice the impact of comorbidities–including atrial fibrillation, abdominal obesity, and BMI at the baseline–on adropin changes in circulation, while there was a relation between ΔBMI and an increase in adropin levels in the female population. Therefore, adropin demonstrated its predictive ability for the improvement of hemodynamics beyond NT-proBNP dynamics. It is a surprise that the tendency of BMI to decline was associated with an increase in adropin, although there are several plausible explanations of this finding. First, ΔBMI might be a surrogate marker of a euvolemic condition, which illustrates the relation of improving hemodynamic performances to decreasing neurohumoral activation [64]. Second, SGLT2 inhibitors may stimulate weight loss through improving glucose and lipid metabolism. Third, a difference in adipose tissue accumulation in females and males may be the indirect gender-related effect of SGLT2 inhibitors on clinical outcomes [29]. Another explanation may relate to the role of enlarged epicardial adipose tissue (EAT) in the regulation of cardiac function. In fact, EAT is not only a conventional cause of the mechanical constriction of the diastolic filling, but it is also a source of pro-inflammatory mediators capable of causing inflammation, microcirculatory dysfunction, and fibrosis of the underlying myocardium, thus impairing the relatability of the LV. Indeed, a recent clinical study revealed that SGLT2 inhibitors have been associated with the attenuation of EAT enlargement [65]. Fifth, SGLT2 inhibitors with anti-inflammatory potency may regulate the synthesis and secretion of adropin by hepatocytes [66]. Finally, SGLT2 inhibitors may increase the circulating levels of adropin indirectly through ketogenesis, and decrease body mass [29,67]. However, the impact of ΔBMI on dynamic changes in adropin in T2DM patients with HF deserves thorough investigation in the future.

Nonetheless, adropin appears to be valuable biomarker that seems to have an additive effect on risk stratification among T2DM patients with HF treated with SGLT2 inhibitors. However, this effect seems to be gender-relative. Indeed, the changes in the adropin levels are regarded to be a predictor, independent of NT-proBNP, for the favorable modification of hemodynamic performances mainly in the female population with T2DM and HF. Perhaps continuous serial measures of adropin may be practically useful for predicting the ability of current HF strategies to reverse cardiac remodeling in both genders, especially taking into consideration of the affordability of the single measure of this biomarker. More studies are required to clearly elucidate the origin of specific mechanisms underlying the association between adropin and the suppression of adverse cardiac remodeling mainly in the female population. Therefore, it would be interesting to use IL-10, adiponectin, or leptin to explain the change in adipose tissue during therapy with SGLT2 inhibitors in connection with the dynamics of adropin.

This study has several limitations, one of which is the open-label design and the lack of a placebo comparison and control group. We understand that a control group is essential for clinical studies, but SGLT2 inhibitors are now generally recommended in HF patients regardless of T2DM presence, so there was no possibility of creating a control group beyond SGLT2 inhibitor administration. However, this is the first study in which the effect of SGLT2 inhibitor dapagliflosin on adropin levels in connection with gender and cardiac remodeling parameters was successfully detected. The next limitation is the lack of measurement of adipose tissue accumulation, which was due to the plausibility of the gender-dependent mechanisms of adropin co-regulation corresponding to body fat composition. This hypothesis deserves to be evaluated in clinical studies in the future. Moreover, we included in the study the patients with HbAc1 < 6.9% who were not treated with insulin to exclude the direct effect of hyperglycemia and insulin therapy on adropin levels. The age of the population studied was relatively young; however, it cannot be ignored that a larger sample could have nullified the differences that emerged between the two genders, particularly with cohorts of older and senior people. All of these factors require further investigation in the future. However, we think these limitations will not intervene in interpreting the results of the study.

## 5. Conclusions

In the population of T2DM patients with HF, mainly in females, the increased circulating levels of adropin seemed to be independent from the NT-proBNP predictor for the favorable modification of hemodynamic performances during the long-term administration of SGLT2 inhibitor dapagliflosin.

## Figures and Tables

**Table 1:** Baseline general characteristics of eligible T2DM patients.

Variables	Entire Patient Population (*n* = 417)	Male Population (*n* = 231)	Female Population (*n* = 186)	*p* Value
Demographics and anthropomorphic parameters				
Age, year	53 (41–64)	52 (40–63)	54 (42–66)	0.262
BMI, kg/m^2^	25.8 ± 2.8	26.4 ± 2.5	25.4 ± 2.2	0.722
Waist circumference, cm	85.1 ± 3.2	86.9 ± 3.0	83.5 ± 2.6	0.180
WHR, units	0.85 ± 0.05	0.87 ± 0.06	0.84 ± 0.04	0.321
Concomitant diseases, comorbidities and CV risk factors				
Dyslipidemia, *n* (%)	346 (83.0)	198 (85.7)	150 (80.6)	0.822
Hypertension, *n* (%)	352 (84.4)	192 (83.1)	160 (86.0)	0.781
Stable CAD, *n* (%)	141 (33.8)	75 (32.5)	66 (35.5)	0.800
Smoking, *n* (%)	168 (40.3)	98 (42.4)	70 (37.6)	0.052
Abdominal obesity, *n* (%)	179 (42.9)	106 (45.9)	73 (39.2)	0.046
Microalbuminuria, *n* (%)	135 (32.4)	79 (34.2)	56 (30.1)	0.120
LV hypertrophy, *n* (%)	334 (80.1)	191 (82.7)	143 (76.9)	0.051
CKD 1–3 grades, *n* (%)	112 (26.9)	62 (26.8)	50 (28.9)	0.822
Paroxysmal/persistence form of atrial fibrillation, *n* (%)	57 (13.7)	34 (14.7)	23 (12.4)	0.120
HF phenotypes and functional classification				
HFpEF, *n* (%)	132 (31.7)	64 (27.7)	68 (36.6)	0.044
HFmrEF, *n* (%)	140 (33.6)	74 (32.0)	66 (35.5)	0.661
HFrEF, *n* (%)	145 (34.8)	93 (40.3)	52 (27.9)	0.042
II/III HF NYHA class, *n* (%)	282 (67.6)/135 (32.4)	143 (61.9)/88 (38.1)	139 (74.7)/47 (25.3)	0.040
Hemodynamics parameters				
SBP, mm Hg	129 ± 6	131 ± 7	129 ± 5	0.864
DBP, mm Hg	78 ± 5	79 ± 6	77 ± 4	0.803
LVEDV, mL	162 (154–170)	166 (159–178)	161 (153–168)	0.040
LVEDVi, mL/m^2^	79 (75–83)	81 (77–87)	78 (75–81)	0.048
LVESV, mL	86 (80–93)	92 (85–98)	83 (80–93)	0.030
LVESVi, mL/m^2^	42 (39–45)	44 (41–48)	40 (38–42)	0.042
LVEF, %	46 (39–54)	44 (37–50)	48 (41–56)	0.050
LVMMI, g/m^2^	154 ± 5	169 ± 6	152 ± 4	0.040
LAVI, mL/m^2^	43 (37–52)	47 (41–56)	39 (36–45)	0.010
E/e’, unit	13.5 ± 0.3	14.9 ± 0.3	12.8 ± 0.2	0.010
Biomarkers				
eGFR, mL/min/1.73 m^2^	75 ± 4.0	73 ± 3.0	76 ± 5.0	0.703
HOMA-IR	7.95 ± 2.3	8.12 ± 2.2	7.81 ± 2.5	0.682
Fasting glucose, mmol/L	5.62 ± 1.3	5.70 ± 1.2	5.60 ± 1.3	0.804
HbA1c, %	6.59 ± 0.02	6.59 ± 0.02	6.58 ± 0.03	0.820
Creatinine, µmol/L	108.6 ± 8.5	112.3 ± 9.3	106.5 ± 7.8	0.240
TC, mmol/L	6.43 ± 0.60	6.60 ± 0.70	6.32 ± 0.55	0.682
HDL-C, mmol/L	0.97 ± 0.17	0.95 ± 0.19	0.99 ± 0.13	0.881
LDL-C, mmol/L	4.38 ± 0.10	4.50 ± 0.12	4.31 ± 0.12	0.860
TG, mmol/L	2.21 ± 0.17	2.27 ± 0.12	2.18 ± 0.15	0.780
NT-proBNP, pmol/mL	2615 (1380–3750)	3218 (1450–4120)	2344 (1296–3901)	0.020
Adropin, ng/mL	2.37 (1.91–2.75)	2.11 (1.82–2.68)	2.69 (2.32–3.04)	0.010
Concomitant medications				
ACEI, *n* (%)	198 (47.5)	106 (45.9)	92 (49.4)	0.880
ARB, *n* (%)	67 (16.1)	34 (14.7)	33 (17.7)	0.760
ARNI, *n* (%)	165 (39.6)	92 (39.8)	73 (39.2)	0.880
Beta-blocker, *n* (%)	372 (89.2)	201 (87.0)	171 (91.9)	0.223
Ivabradine, *n* (%)	59 (14.1)	32 (13.9)	27 (14.5)	0.864
Calcium channel blocker, *n* (%)	75 (18.0)	38 (16.5)	37 (19.9)	0.481
MRA, *n* (%)	283 (67.8)	150 (64.9)	133 (71.5)	0.053
Loop diuretic, *n* (%)	358 (85.9)	196 (84.8)	162 (87.1)	0.871
Antiplatelet, *n* (%)	367 (88.0)	201 (87.0)	166 (89.2)	0.882
Anticoagulant, *n* (%)	51 (12.2)	34 (14.7)	17 (9.1)	0.040
Metformin, *n* (%)	387 (92.8)	210 (90.9)	177 (95.2)	0.623
Statins, *n* (%)	408 (97.8)	226 (97.9)	182 (97.8)	0.990

Abbreviations: ACEI, angiotensin-converting enzyme inhibitor; ARNI angiotensin receptor neprilysin inhibitor; CAD, coronary artery disease; CKD, chronic kidney disease; BMI, body mass index; DBP, diastolic blood pressure; E/e’, early diastolic blood filling to longitudinal strain ratio; GFR, glomerular filtration rate; HDL-C, high-density lipoprotein cholesterol; HFpEF, heart failure with preserved ejection fraction; HFmrEF, heart failure with mildly reduced ejection fraction; HFrEF, heart failure with reduced ejection fraction; LVEDV, left ventricular end-diastolic volume; LVEDVi, left ventricular end-diastolic volume index; LVESV, left ventricular end-systolic volume index; LVEF, left ventricular ejection fraction; LVMMI, left ventricle myocardial mass index; LAVI, left atrial volume index; LDL-C, low-density lipoprotein cholesterol; MRA, mineralocorticoid receptor antagonist; SBP, systolic blood pressure; TG, triglycerides; TC, total cholesterol; WHR, waist-to-hip ratio. Notes: data of variables are given as mean ± SD and median (25%−75% interquartile range).

**Table 2:** Comparisons between baseline and 6-month variables after the administration of SGLT2 inhibitor dapagliflozin in males and females.

Variables	Baseline	Over 6 Months	Δ%	*p* Value
Clinical characteristics				
BMI, kg/m^2^	25.8 ± 2.8	24.1 ± 1.9	−4.30	0.113
Male	26.4 ± 2.5	25.3 ± 1.7	−4.10	0.142
female	25.4 ± 2.2	23.9 ± 1.8	−5.90	0.104
II HF NYHA class, *n* (%)	282 (67.6)	329 (78.9)	+14.3	0.040
Male	143 (50.7)	154 (46.8)	+11.0	0.040
female	139 (49.3)	175 (53.2)	+36.0	0.010
III HF NYHA class, *n* (%)	135 (32.4)	88 (21.1)	−34.8	0.040
Male	88 (65.2)	77 (87.5)	−12.5	0.040
female	47 (34.8)	11 (12.5)	−76.5	0.001
Hemodynamics performances				
LVEDV, mL	162 (154–170)	158 (150–167)	−1.90	0.460
Male	166 (159–178)	165 (156–173)	−0.60	0.904
female	161 (153–168)	156 (151–162)	−3.10	0.061
LVESV, mL	86 (80–93)	80 (76–85)	−7.00	0.040
Male	92 (85–98)	87 (83–93)	−5.40	0.052
female	83 (80–93)	75 (71–79)	−9.60	0.020
LVEF, %	46 (39–54)	50 (44–57)	+8.60	0.054
Male	44 (37–50)	47 (39–54)	+6.80	0.160
female	48 (41–56)	52 (48–59)	+8.70	0.050
LVMMI, g/m^2^	154 ± 5	141 ± 5	−8.40	0.020
Male	169 ± 6	155 ± 7	−8.20	0.050
female	152 ± 4	138 ± 4	−9.20	0.010
LAVI, mL/m^2^	39 (34–45)	35 (31–39)	−10.3	0.040
Male	47 (41–56)	43 (37–49)	−8.5	0.050
female	39 (36–45)	34 (32–37)	−12.8	0.010
E/e’, unit	13.5 ± 0.3	10.7 ± 0.5	−20.7	0.020
Male	14.9 ± 0.3	12.6 ± 0.4	−15.4	0.040
female	12.8 ± 0.2	9.88 ± 0.3	−22.8	0.020
Biomarkers				
eGFR, mL/min/1.73 m^2^	75 ± 4.0	78 ± 3.0	+4.0	0.820
male	73 ± 3.0	76± 4.0	+3.9	0.842
female	76 ± 5.0	80± 5.0	+5.2	0.813
Fasting glucose, mmol/L	5.62 ± 1.3	4.90 ± 1.0	−12.8	0.240
male	5.70 ± 1.2	4.95 ± 1.1	−13.1	0.240
female	5.60 ± 1.3	4.94 ± 1.2	−11.7	0.260
HbA1c, %	6.59 ± 0.02	6.47 ± 0.03	−1.74	0.312
male	6.59 ± 0.02	6.49 ± 0.03	−1.50	0.263
female	6.58 ± 0.03	6.46 ± 0.03	−1.80	0.204
Creatinine, µmol/L	108.6 ± 8.5	112.5 ± 7.0	+3.50	0.282
male	112.3 ± 9.3	116.8 ± 8.5	+3.80	0.404
female	106.5 ± 7.8	109.8 ± 8.0	+3.10	0.361

Notes: data of variables are given as mean ± SD and median (25−75% interquartile range). Abbreviations: E/e’, early diastolic blood filling to longitudinal strain ratio; HF, heart failure; HbA1c, glycosylated hemoglobin; GFR, glomerular filtration rate; LVESV, left ventricular end-systolic volume; LVEF, left ventricular ejection fraction; LVMMI, left ventricle myocardial mass index; LAVI, left atrial volume index; Δ%, respective percentage of changes of parameters.

**Table 3:** Univariate and multivariate linear regression analyses of the association of adropin levels with age, abdominal obesity, BMI, NYHA class, and relative changes in hemodynamics and NT-proBNP.

Variables	Univariate Linear Regression			Multivariate Linear Regression		
	B Coefficient	SD	*p* Value	B Coefficient	SD	*p* Value
Entire patient cohort						
Age	0.73	0.30	0.290	-		
Obesity	0.90	0.25	0.05	0.71	0.20	0.060
BMI at baseline	1.10	0.38	0.046	0.66	0.25	0.104
ΔBMI	−1.64	0.29	0.055	−1.65	0.22	0.052
NYHA class	−1.92	0.35	0.042	−1.36	0.24	0.052
ΔLVESV	−2.10	0.72	0.053	−1.99	0.52	0.120
ΔLVEF	3.26	0.42	0.040	2.73	0.50	0.046
ΔLVMMI	−2.61	1.20	0.012	−2.40	1.14	0.052
ΔLAVI	−6.15	1.60	0.001	−6.10	1.54	0.001
ΔE/e’	−7.90	1.26	0.001	−7.83	1.22	0.001
ΔNT-proBNP	−3.22	1.92	0.012	−2.06	0.66	0.050
Male subgroup						
Age	0.75	0.33	0.266	-		
Obesity	0.90	0.27	0.05	0.66	0.25	0.104
BMI at baseline	1.10	0.38	0.046	1.02	0.29	0.216
ΔBMI	−1.29	0.22	0.162	-		
NYHA class	−0.68	0.20	0.640	-		
ΔLVESV	−1.23	0.55	0.600	-		
ΔLVEF	2.01	0.17	0.120	-		
ΔLVMMI	−2.42	0.98	0.050	−2.40	1.14	0.054
ΔLAVI	−5.80	1.20	0.042	−2.30	1.17	0.050
ΔE/e’	−7.30	1.50	0.012	−5.90	1.70	0.014
ΔNT-proBNP	−3.10	1.68	0.014	−2.10	1.52	0.060
Female subgroup						
Age	0.71	0.26	0.293	-		
Obesity	0.90	0.27	0.048	0.78	0.22	0.062
BMI at baseline	1.12	0.31	0.041	1.06	0.31	0.055
ΔBMI	−1.76	0.27	0.042	−1.70	0.23	0.044
NYHA class	−1.92	0.35	0.042	−1.36	0.24	0.050
ΔLVESV	−2.10	0.72	0.053	−1.99	0.52	0.121
ΔLVEF	3.26	0.42	0.040	2.73	0.50	0.046
ΔLVMMI	−2.61	1.20	0.001	−2.40	1.14	0.050
ΔLAVI	−6.15	1.60	0.001	−6.10	1.54	0.001
ΔE/e’	−7.90	1.26	0.001	−7.83	1.22	0.001
ΔNT-proBNP	−1.22	0.92	0.012	−1.06	0.66	0.050

Abbreviations: BMI, body mass index; SD, standard deviation; NYHA, New York Heart Association; Δ, a relative change in variables after 6-month administration of dapagliflozin.

## Data Availability

Not applicable.
